# Multimodal Stimulation of Colorado Potato Beetle Reveals Modulation of Pheromone Response by Yellow Light

**DOI:** 10.1371/journal.pone.0020990

**Published:** 2011-06-10

**Authors:** Fernando Otálora-Luna, Joseph C. Dickens

**Affiliations:** 1 Laboratorio de Ecología Sensorial, Centro de Estudios Interdisciplinario de la Física (CEIF), Instituto Venezolano de Investigaciones Científicas (IVIC), Loma de Los Guamos, Parroquia Jají, Estado Mérida, República Bolivariana de Venezuela; 2 Invasive Insect Biocontrol and Behavior Laboratory, Plant Sciences Institute, Henry A. Wallace Beltsville Agricultural Research Center, Agricultural Research Service, United States Department of Agriculture, Beltsville, Maryland, United States of America; Ghent University, Belgium

## Abstract

Orientation of insects to host plants and conspecifics is the result of detection and integration of chemical and physical cues present in the environment. Sensory organs have evolved to be sensitive to important signals, providing neural input for higher order multimodal processing and behavioral output. Here we report experiments to determine decisions made by Colorado potato beetle (CPB), *Leptinotarsa decemlineata*, in response to isolated stimuli and multimodal combinations of signals on a locomotion compensator. Our results show that in complete darkness and in the absence of other stimuli, pheromonal stimulation increases attraction behavior of CPB as measured in oriented displacement and walking speed. However, orientation to the pheromone is abolished when presented with the alternative stimulation of a low intensity yellow light in a dark environment. The ability of the pheromone to stimulate these diurnal beetles in the dark in the absence of other stimuli is an unexpected but interesting observation. The predominance of the phototactic response over that to pheromone when low intensity lights were offered as choices seems to confirm the diurnal nature of the insect. The biological significance of the response to pheromone in the dark is unclear. The phototactic response will play a key role in elucidating multimodal stimulation in the host-finding process of CPB, and perhaps other insects. Such information might be exploited in the design of applications to attract and trap CPB for survey or control purposes and other insect pests using similar orientation mechanisms.

## Introduction

The Colorado potato beetle (CPB), *Leptinotarsa decemlineata* (Say), (Coleoptera: Chrysomelidae) is one of the most important pests in Eurasia and North America and an appreciated model for insect behavioral studies. CPB lives in association with potato crop or other solanaceous plants where feeding and oviposition occur. Several hours following emergence, adults begin to search for food. After a few days their reproductive system and flight muscles complete development at which time beetles search for mating partners [1].

As with many other phytophagous insects, CPB relies mainly on visual and olfactory information to find host plants and conspecifics [2]–[5]. There is increasing interest in understanding CPB behavior in order to develop novel strategies and pesticide alternatives for economic management of this pest [6], [7], and to shed light on the principles that are the bases of the *sensory ecology* [8] of this model insect.

The attractancy to colors, especially yellow, to arthropods is well known and has been broadly reported for many phytophagous insects [9], [10]. In CPB, a yellow stimulus elicits electrophysiological responses from visual receptors in compound eyes [11], [12] as well as orientation behaviors [5], [13], [14]; surfaces colored with yellow, as well as orange and green pigments, are more attractive than similar white, blue, red or black surfaces. However, reproducibility and characterization of spectra is difficult to achieve using pigmented surfaces, because these surfaces reflect colors that vary depending on the spectral composition of the illuminating light. Thus other researchers have now used light emitting diodes (LEDs) to test color preferences [15]. Recently, these authors used a dual-choice arena adapted to a locomotion compensator to demonstrate that yellow (585 nm) light emitted by LEDs is more attractive to CPB than similar white (420 nm–775 nm), ultraviolet (351 nm), blue (472 nm), orange (590 nm) and red (660 nm) lights.

Sex and aggregation pheromones have been demonstrated for many insects [16] including CPB [17]. The male-produced aggregation pheromone (*S*)-CPB I elicits electrophysiological responses from olfactory receptors on the antenna of adult CPB and is a potent attractant in dual choice behavioral bioassays under laboratory conditions [18]. This pheromone also elicits attraction of CPB larvae on a servosphere [19]. Additionally, this molecule has been demonstrated to be an effective lure in field tests [20]. While an initial synthesis of (*S*)-CPB I was reported [21]; recently an economic route to its production was published [22].

These studies and other laboratory observations have shown that CPB and other arthropods [23] can orient to a particular goal (e.g. food, mate or refuge) by using a single sensory modality. However, environmental information is usually redundant, and in nature CPB and other phytophagous animals must decipher and respond to multiple sensory cues when orienting to host plants and mates. Knowledge of how arthropods behave when offered a multimodal stimulus is limited. There are a few reports of a synergistic behavioral effect on naïve insects stimulated with relevant visual and olfactory cues [24], [25]. Some authors have found an improvement of insect learning when two modalities are combined [26]. Research on how naïve arthropods respond when offered choices between two or more relevant signals from different modalities is also scarce [27], [28], [29], [30]. 

**Figure 1:**
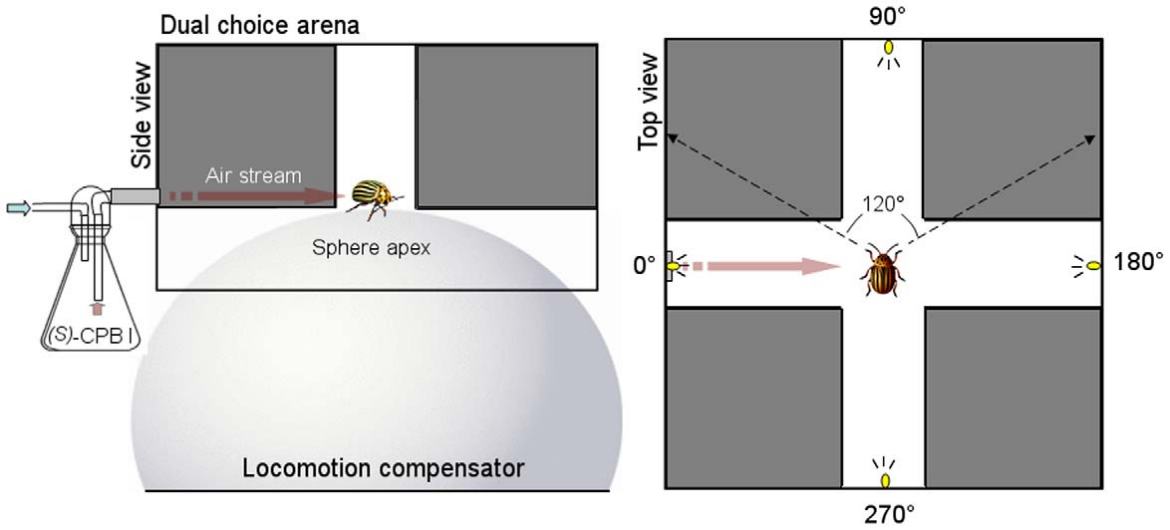
Dual choice arena adapted to servosphere. Schematic representation of dual choice arena adapted to the locomotion compensator where an *L. decemlineata* beetle walking on the apex of a sphere was stimulated with a male-produced aggregation pheromone (*S*)-CPB in an air stream (side view) and opposite yellow LEDs (585 nm). The insect was able to perceive the stimuli but unable to reach any of the four arms (top view). Relative distance walked in arbitrary cones, 60° either side of 0°, 90°, 180° and 270° directions respectively, served to quantify beetle preferences (see dashed vectors).

In order to understand the relative importance of olfaction in the presence of light, CPB that had both aggregation and nutritional needs were exposed to an aggregation pheromone and an attractive yellow light stimulus. We first studied orientation of naïve beetles stimulated with the male-produced aggregation pheromone (*S*)-CPB I in complete darkness. Choices between the two attractive stimuli were also offered simultaneously: the (*S*)-CPB I pheromone and yellow light in a dual choice arena, where the yellow light was the only source of illumination (Fig. 1). We tested if the pheromone was attractive in the presence of the yellow lights when the stimuli were offered in different corridors of our behavioral chamber. Finally, we tested if the insect oriented preferably to a source of pheromone plus yellow light over yellow light alone. By using a locomotion compensator, we could measure orientation and walking speed of both sexes to the various stimulus paradigms. The biological significance of the our results are discussed with regard to the diurnal nature of the insect and may play a key role in elucidating behavioral responses to multimodal stimuli in CPB, and perhaps other insects [19].

## Results

**Figure 2:**
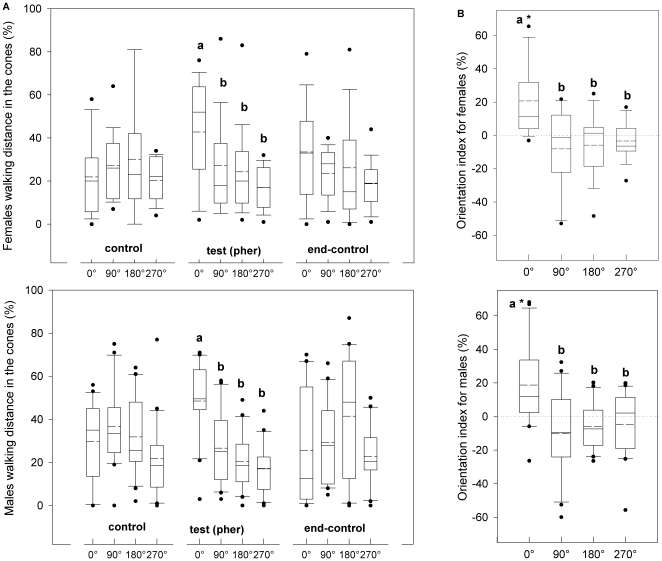
Orientation of Colorado potato beetle to aggregation pheromone in darkness. Orientation behaviors of female and male CPBs stimulated with a male-produced aggregation pheromone (*S*)-CPB I in an air stream (pher) **a.** Proportion of distance covered during the three experimental periods (control, test and end-control) in each of four 120° cones projected to 0°, 90°, 180° and 270° respectively. **b.** Orientation index calculated as the proportion of distance covered in each cone during the test period minus the proportion of the distance covered in each cone during the first control period. Asterisks (*) indicate means that are significantly different from zero (Wilcoxon signed rank test, p<0.05). Different letters indicate significant pairwise differences across the different directions (Dunn's post-hoc test, p<0.05). In the boxplots, the *line* within a box mark the median, the *segmented line* within a box mark the mean, the *lower and upper boundary lines* of a box indicate the 25^th^ and 75^th^ percentiles, *bars below and above* indicate the 10^th^ and 90^th^ percentiles, respectively, and the *points* represent data beyond these limits.

The arrangement of stimulus sources offered to individual CPB during experiments is illustrated in Figure 1. In the absence of light, the addition of the aggregation pheromone (*S*)-CPB I to the air stream during the test period induced both female and male CPB to walk more upwind (0°) towards the source of the chemo-stimulation than either crosswind (90° and 270°) or downwind (180°) (Fig. 2a). Walking distances in the 120° cones assigned to each of the four arena arms (see Materials and Methods) were significantly different between the four directions during the test period (Kruskal-Wallis test, p<0.01), but were not significantly different during the control and end-control periods (Kruskal-Wallis test, p>0.05, Fig. 2a). Both sexes walked more upwind during the test period compared to the previous control period (i.e. upwind indexes are significantly higher than zero, Fig. 2b). Orientation indexes were significantly different between the four directions for both sexes (Kruskal-Wallis test, p<0.01). No significant differences were found between orientation of females and males (Wilcoxon unpaired test, p>0.05).

**Figure 3:**
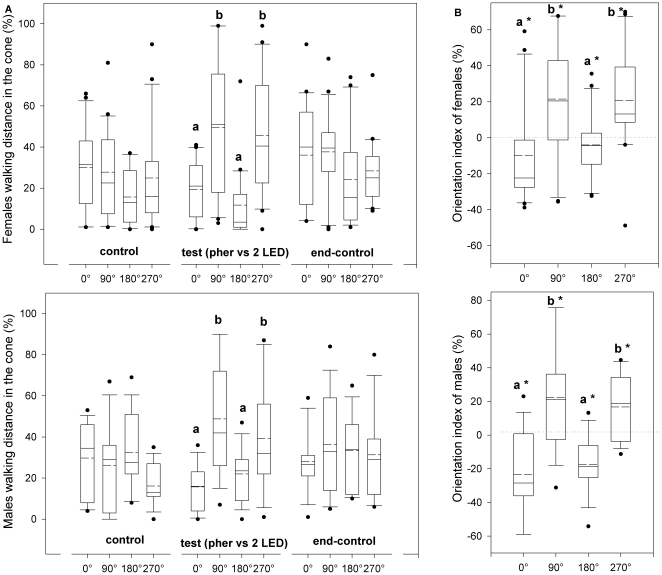
Orientation of Colorado potato beetle to light vs. aggregation pheromone. Orientation behaviors of female and male CPBs stimulated simultaneously with a male produced aggregation pheromone (*S*)-CPB I in an air stream and two opposite LED beams of yellow light perpendicular to the air stream (pher vs. 2 LED). **a.** Proportion of distance covered during the three experimental periods (control, test and end-control) in each of four 120° cones projected to 0°, 90°, 180° and 270° respectively. **b.** Orientation index calculated as the proportion of distance covered in each cone during the test period minus the proportion of the distance covered in each cone during the first control period. Asterisks (*) indicate means that are significantly different from zero (Wilcoxon signed rank test, p<0.05). Different letters indicate significant pairwise differences across the different directions (Dunn's post-hoc test, p<0.05). In the boxplots, the *line* within a box mark the median, the *segmented line* within a box mark the mean, the *lower and upper boundary lines* of a box indicate the 25^th^ and 75^th^ percentiles, *bars below and above* indicate the 10^th^ and 90^th^ percentiles, respectively, and the *points* represent data beyond these limits.

When female and male CPB were simultaneously stimulated with the pheromone and two opposite yellow LEDs perpendicular to the air stream during the test period, both sexes walked more crosswind, i.e. toward the yellow lights (Fig. 3a). Walking distances in the cones were significantly different between the four directions during the test period (Kruskal-Wallis test, p<0.01), but were not significantly different during the initial control and end-control periods (Kruskal-Wallis test, p>0.05, Fig. 3a). Both sexes walked more crosswind and less upwind and downwind during the test period compared to the previous control period (i.e. crosswind indexes were significantly higher than zero and upwind and downwind indexes were significantly lower than zero, Fig. 3b). Orientation indexes were significantly different between the four directions for both sexes (Kruskal-Wallis test, p<0.05). No significant differences were found between orientation of females and males (Wilcoxon unpaired test, p>0.05).

**Figure 4:**
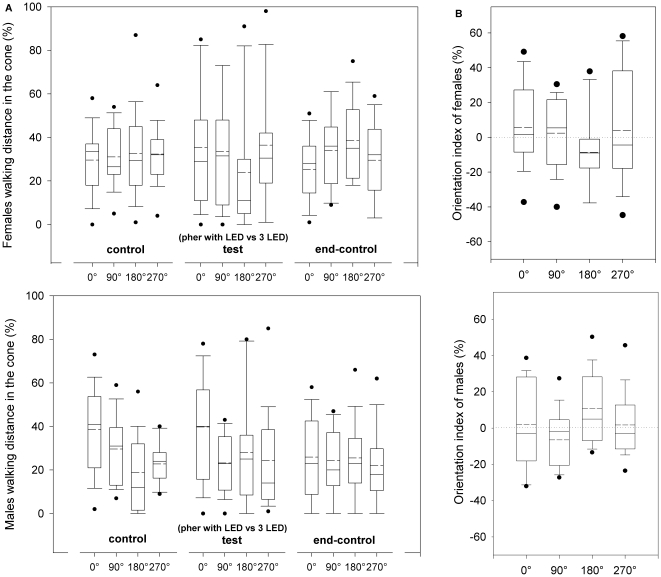
Orientation of Colorado potato beetle to aggregation pheromone in the presence of yellow light. Orientation behaviors of female and male CPBs stimulated simultaneously with a male produced aggregation pheromone (*S*)-CPB I in an air stream and four opposite LED beams of yellow light, two perpendicular and two parallel to the air stream (pher vs. 4 LED). **a.** Proportion of distance covered during the three experimental periods (control, test and end-control) in each of four 120° cones projected to 0°, 90°, 180° and 270° respectively. **b.** Orientation index calculated as the proportion of distance covered in each cone during the test period minus the proportion of the distance covered in each cone during the first control period. In the boxplots, the *line* within a box mark the median, the *segmented line* within a box mark the mean, the *lower and upper boundary lines* of a box indicate the 25^th^ and 75^th^ percentiles, *bars below and above* indicate the 10^th^ and 90^th^ percentiles, respectively, and the *points* represent data beyond these limits.

When female and male CPB were simultaneously stimulated with the pheromone plus one yellow LED (0°) versus the other three yellow LEDs (90°, 180° and 270°) during the test period, insects did not show preference for any direction (Fig. 4a). Walking distances in the cones were not significantly different between the four directions during the control, test or end-control periods (Kruskal-Wallis test, p>0.05, Fig. 4a). Walking distance in the cones did not differ between the test and the previous control period (i.e. indexes are not significantly different than zero, Fig. 4b). Orientation indexes were not significantly different between the four directions for either sex (Kruskal-Wallis test, p<0.05). No significant differences were found between orientation of females and males (Wilcoxon unpaired test, p>0.05).

**Figure 5:**
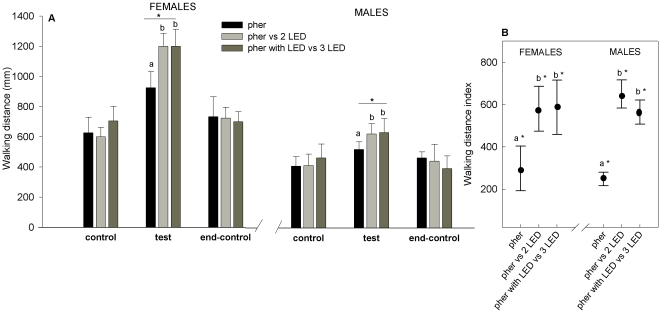
Walking distance of Colorado potato beetle is increased by light and pheromone. Distance walked by CPBs during stimulation with: the pheromone alone (pher), the pheromone versus two LED beams of yellow light perpendicular to the air stream (pher vs. 2 LED) and the pheromone plus one LED versus three opposite LED beams of yellow light: two perpendicular and two parallel to the air stream (pher with LED vs. 3 LED). **a.** Average walking distance during the three experimental periods (control, test and end-control) of females and males. Asterisks (*) over the horizontal lines indicate that distances were significantly different between the three stimulation treatments (one-way ANOVA, p<0.01). **b.** Walking distance index was calculated as the average walking distance during the test period minus the average walking distance during the first control period. Asterisks (*) indicate means that are significantly different from zero (Paired t-test, p<0.05). Different letters indicate significant pairwise differences across the different directions (Tukey-Kramer's post-hoc tests, p<0.05). The *black filled circles* mark the mean and *bars below and above* indicate the standard error.

Female CPB tested during the control periods (i.e. naïve insects) walked an average distance of 604.0 mm (SD 314.1 mm, n = 66) and males an average distance of 439.3 mm (SD 271.6 mm, n = 66). During the test period both sexes increased their walking distance in response to the three different stimulus treatments: the pheromone alone, the pheromone plus the two yellow LEDs, and the pheromone plus the four opposite yellow LEDs (Fig. 5). Walking distances of both sexes were increased significantly by each stimulus treatment (one-way ANOVA, P<0.01). Walking distances of both sexes decreased after most stimulus treatments, i.e. during the end-control period compared with the test period (repeated measures ANOVA, P<0.05). Insects walked longer distances during the test period when compared to the prior control period (i.e. walking distance indexes were significantly higher than zero, Fig. 5b). Walking distance indexes were significantly different between the three stimulus treatments (one-way ANOVA, p<0.01); insects walked more during stimulation with pheromone plus yellow light than with pheromone alone (Fig. 5a,b). Females walked more than males during control, test and end-control periods (unpaired t-test, p<0.05, Fig 5a,b).

**Figure 6:**
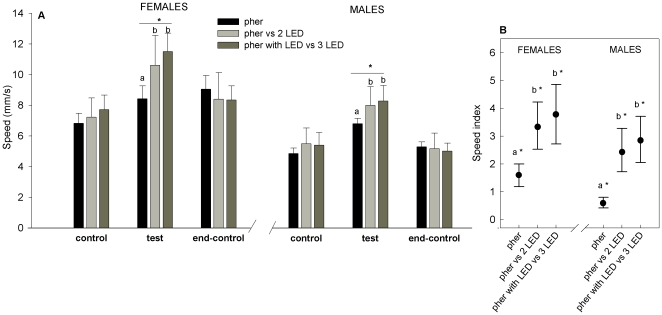
Walking speed of Colorado potato beetle is increased by light and pheromone. Speed reached by CPBs during stimulation with: the pheromone alone (pher), the pheromone versus two LED beams of yellow light perpendicular to the air stream (pher vs. 2 LED) and the pheromone plus one LED versus three opposite LED beams of yellow light: two perpendicular and two parallel to the air stream (pher with LED vs. 3 LED). **a.** Average speed during the three experimental periods (control, test and end-control) of females and males. Asterisks (*) over the horizontal lines indicate that velocities were significantly different between the three stimulation treatments (one-way ANOVA, p<0.01). **b.** Speed index was calculated as the average speed during the test period minus the average speed during the first control period. Asterisks (*) indicate means that are significantly different from zero (Paired t-test, p<0.05). Different letters indicate significant pairwise differences across the different directions (Tukey-Kramer's post-hoc tests, p<0.05). The *black filled circles* mark the mean and *bars below and above* indicate the standard error.

Female CPB tested during the control periods (i.e. naïve insects) walked at an average speed of 7.4 mm/s (SD 2.0 mm/s, n = 66) and males at an average speed of 5.1 mm/s (SD 1.8 mm/s, n = 66). During the test period, both sexes increased their speed in response to the three different stimulus treatments (Fig. 6). The maximum speed recorded (during photic stimulation) for females was 20.7 mm/s and for males 12.9 mm/s. Velocities of both sexes were increased significantly by each stimulus treatment (one-way ANOVA, P<0.01). The velocities of both sexes decreased after most stimulus treatments, i.e. during the end-control period compared with the test period (Repeated measures ANOVA, P<0.05), except for the speed of females for ‘pheromone alone’ that did not decrease (Repeated measures ANOVA, P>0.05, Fig. 6a). Velocities were greater during the end-control period for all females treatments compared to the initial control period (Friedman's ANOVA between the two control periods, P<0.05), but velocities of males returned to pre-stimulation levels during the end-control period (P>0.05, Fig. 6a). When considering classes categories separately, we also found significant speed differences between the initial control period and the end-control period for females (paired t-test, P<0.05), but not for males (P>0.05). Insects walked faster during the test period when compared to the prior control period (i.e. speed indexes were significantly higher than zero, Fig. 5b). Speed indexes were significantly different between the three stimulus treatments (one-way ANOVA, p<0.01); insects walked faster during stimulation with pheromone plus yellow light than with pheromone alone (Fig. 6a,b). Females walked faster than males during control, test and end-control periods (unpaired t-test, p<0.05, Fig 6a,b).

## Discussion

In the present study, three different sensory modalities were stimulated: one associated with the yellow light (i.e. photoreception) and two associated with the pheromone odor (i.e. olfaction and mechanoreception). In nature, mechanoreception is usually implied in olfaction, as odors are carried by air currents detected by mechanoreceptors. In our current study, the pheromone was carried by an air current that served as an eolic cue that provided information to the CPB associated with the direction of the chemical source by stimulating its mechanoreceptors.

The orientation of CPB to the male-produced aggregation pheromone (*S*)-CPB I in darkness was unexpected. Previously, we showed that CPB orientated to the pheromone in the presence of white light; an observation which may correlate with the diurnal nature of the insect [18]. While the source dose used in the present study was 10 µg, the effective amount of pheromone reaching the insect was likely much lower than the highest dose (10 µg) used in the earlier study [18]. Here air passing through a bottle containing the odor was diluted 40-fold before reaching the insect (see Materials and Methods), while in the earlier study air carried the odor from the source directly to the insect. In that study, a dual choice “T-track olfactometer” was used where the insects climb to the top of a copper “T” illuminated with an incandescent light source. The light induced the beetles to climb up the copper “T”; subsequently having to choose between lateral sources of pheromone-laden or clean air. Thus, light could have influenced chemotactic responses as has been observed in other beetles and moths [27], [31]. While the biological significance of the response of CPB to the pheromone in darkness is unclear, our results suggest that CPB may be active at night and respond to the pheromone.

CPB phototactic behavior has been previously observed for walking [32] and flying [33] (nondiapausing and prediapausing) adults stimulated with white light. Yellow light was selected among different wavelengths for this study because it has proven to be highly attractive to CPB in previous experiments, even more attractive than white light [15]. When the pheromone was offered simultaneously with this light, orientation of both sexes to the male-produced pheromone was diminished as most of insects walked crosswind toward the light stimulus. This result is surprising as previous dual choice laboratory bioassays performed under different light conditions show the pheromone to be highly attractive. However, the predominance of the phototactic response over that to pheromone when low intensity lights were offered as choices seems to confirm the diurnal nature of the insect.

Priority of visual over olfactory cues has been previously reported for other diurnal insects. For example, naïve butterflies such as *Vanessa indica* (Lepidoptera: Nymphalidae) depended primarily on color and secondarily on scent during flower visitation [28]. The diurnal moth *Macroglossum stellatarum* (Lepidoptera: Sphingidae) strongly favored a visual stimulus when both were presented simultaneously [29]. On the other hand, the nocturnal sphingid, *Deilephila elpenor,* responded preferably to odor [29]. Although, the beetle *Trypodendron lineatum* (Coleoptera: Scolytidae) shows phototactic behavior prior to flight experience, such photopositive response is abolished after beetles experience flight, leading to a switch to chemotactic orientation [31].

We used mature adults which had both nutritional and sexual needs; they were looking for mates and food, and probably other resources such as conspecifics and refuge. The highly specific stimulus we used is an aggregation one, produced by males to attract females and males, but that also can attract larvae (i.e. sexually immature individuals), as demonstrated previously using a servosphere [19]. Perhaps insight into such strongly phototactic behavior of chemically stimulated CPB may be provided when the amount of information supplied by vision and olfaction is compared, and when one considers the diurnal nature of the insect. Indeed, the information transmission capacity of complex eyes is estimated to be several orders of magnitude higher compared with the olfactory system [8]. The more information an organism can obtain from the environment, the more possibilities it will have to find resources for survival and reproduction.

Although both sexes increased their speed in response to pheromone and light during the test period, insects walked faster during stimulation with pheromone plus yellow light than with pheromone alone. Probably CPB reaches higher speeds in the presence of light because, as it was mentioned before, this stimulus gives more spatial information that facilitates its movement. Females walked faster than males for all stimulation treatments and during the three experimental periods (control, test and end-control). Such speed differences between sexes can be exclusively attributed to the fact that there is a sexual size dimorphism, i.e. females are generally about 1.21 times larger than males (147±3 mg and 119±2 mg, respectively) [34], [35].

In conclusion, our results show that in complete darkness and in the absence of other stimuli, pheromonal stimulation increases attraction behavior of CPB as measured in oriented displacement and walking speed. However, orientation to the pheromone is diminished when presented with the alternative stimulation of a low intensity yellow light in the otherwise dark environment. The ability of the pheromone to stimulate these diurnal beetles in the dark in the absence of other stimuli was unexpected. While the biological significance of the response to pheromone in the dark is unclear, it suggests that CPB may be active and respond to the pheromone in darkness in the field. A more detailed knowledge of behavioral responses of insects, such as presented here for CPB, to multimodal stimuli enhances our understanding of potential unexpected nuances of behavioral choices and might be exploited in the design or improvement of devices for survey or control of insect pests.

## Materials and Methods

### Insects

Adult Colorado potato beetles (CPB), *Leptinotarsa decemlineata,* were obtained from a laboratory colony that was annually infused with wild insects from Beltsville, MD, USA (39°1′30.21′ ´N, 76°55′31.73′ ´W). Newly emerged adults were sexed and kept separately, to prevent mating, in groups of 10 individuals in plastic containers (height 8 cm, diameter 11 cm) that were placed in a chamber at 25±2°C and 60±5% relative humidity (r.h.). Illumination during the light phase of the L16:D8 cycle was provided by a fluorescent lamp (FG12180-A, General Electric, Fairfield, CT, USA). Long-day conditions assured that insects did not enter diapause. Insects were fed foliage from potato plants, *Solanum tuberosum*, L., cv. Kennebeck (Solanaceae) that were grown in a green house at 25°C and 60±5% r.h. Larvae were fed directly on plants that had been transferred from the greenhouse to the incubators. Twigs severed from greenhouse potato plants and kept at 8°C in plastic bags with a moist cloth until use served to feed adults. Adults used for experiments were 5–14 days post-emergence; at this time, sclerotization of the cuticle, capacity to feed, and sexual maturation [4] were completed. Prior to behavioral experiments, insects were starved for approximately 24 hours. Each individual was used only once.

### Servosphere

An experimental animal was tracked while walking on a locomotion compensator (Syntech LC-300, Hilversum, The Netherlands) or servosphere as previously described [36], [19]. This tracking system allowed the untethered insect to walk unimpeded in all directions on the apex of a 30 cm diameter white sphere. A movement detector based on active pixel sensor (APS) technology integrated with a near-infrared 8-LED (light-emitting diode) lamp (wavelength peak at 940 nm) was positioned 22 cm above the insect. The near-infrared lamp as integrated with the sensor illuminated a field of 7 cm in diameter on the apex of the sphere. The signal from the movement detector was processed and sent to servo-motors that drove the sphere in the opposite direction of the insect's movement in order to maintain the insect's location at the apex of the sphere. Movement of the sphere were supplied to a computer by two pulse-generator encoders positioned orthogonally at the equator of the ball, allowing the reconstruction of tracks described by the insect's movements. The movements were measured at a rate of 0.1/s with an accuracy of 0.1 mm.

### Dual choice arena

The observation system used in this study was tested previously with CPB [15] and consisted of a modified version of a crossroad arena [37]. It comprised two perpendicular corridors (length 36 cm, wide 7 cm, height 14 cm) made of acrylic plastic (polymethyl methacrylate) walls (thick 5 mm) (Precision Plastics Inc, Beltsville, MD, USA). The two corridors bisected each other forming a cross with a square chamber at the center of the servosphere (Fig. 1 A). This central chamber overlapped with the apex of the sphere (Fig. 1 A., side view) and the field of view of the video sensor. The walking insect always rested in this chamber where it was exposed to the experimental stimuli but unable to reach any of the four arms (open loop set up). The inner lateral walls of the arena corridors were covered with steel metal mirrors to avoid biases caused by shadows produced at the corners of the corridors (Fig. 1 A, top view). The top of each arm was covered with a black roof made of acrylic plastic. The central chamber top was left uncovered in order to track the insect movement with the video sensor.

### Chemical stimulus

Insects were stimulated with the male-produced aggregation pheromone (*S*)-3,7-dimethyl-2-oxo-oct-6-ene-1,3-diol [(*S*)-CPB I] [18] during the two minute (test period, see below). (*S*)-CPB I was 96.5% optically pure [21]. The odor delivery system consisted of a charcoal-filtered air-stream maintained at 25°C and 60–73% r.h. as measured by a hygro-thermometer (EA25, Extech Instruments, Waltham, MA, USA; accuracy: ±3% r.h. and ±1°C) flowing at 6 l/min (30±15 cm/s airspeed as measured by a hot-wire anemometer, model 441S, Kurtz, CA, USA; accuracy ±0.01 m/s) that passed through a stainless steel tube (dia. 2 cm, length 4 cm). This tube was painted black, to exclude unexpected influences from asymmetries, and positioned on the horizontal plane tangent to the apex of the sphere and 18 cm from the insect at the end of a corridor of the dual choice arena. A laminar flow over the insect was made by passing the airflow through a spongy steel insert within the tube. A second charcoal-filtered air-stream (Stimulus Controller CS-55, Syntech, The Netherlands) passing through a 500 ml glass bottle containing the pheromone odor source was injected at 150 ml/min immediately downstream of the spongy steel, so the air from the flasks was diluted 40-fold in the main air stream. The pheromone (*S*)-CPB I was applied to a filter paper disc (dia. 90 mm, Whatman No. 1, England) placed at the bottom of the gas-wash bottle. The filter paper was treated with 100 µL of a 0.1 µg/µL (*S*)-CPB I solution of hexane (purity 99.7%, Fluka, USA) to give 10 µg of pheromone on the paper. The hexane solvent was left to evaporate under a hood for approximately 20 s at room temperature prior to placing the filter paper in the flask. The control consisted of a gas-wash bottle loaded with the same amount of solvent on filter paper. The headspace of the control bottles was injected as described previously during the control periods. The flasks were then left to equilibrate for at least 10 min before presenting the chemical stimulus to the beetles. Filter papers containing the pheromone were replaced after 10 test insects.

### Light stimulus

Insects were stimulated with yellow (subjective visual sensation produced on humans) opposite light emitting diodes or LEDs (LED supply, Randolph, VT, USA) during a two minute test period. Yellow emitted by the LEDs had a wavelength band of approximate 20 nm, with a maximum peak at 585 nm and a centroid wavelength of 583 nm, thus the wavelength shape was highly symmetric [15]. The spectral bandwidth range was calculated from the two wavelengths on either side of the spectrum given at the intensity value that equals ½ of the peak value. The beam radiation pattern corresponded to a narrow-angled specular LED. The optical axes of the yellow beams were directed to the insect that walked on the apex of the sphere by positioning LEDs at an angle of 9 degrees to the insect at the end of each of the four corridor arms. The intensity of light emitted by LEDs was regulated with a stimulator (Grass S44 and S88, Grass Instruments, Quincy, MA, USA), so each yellow LED emitted a photon intensity or flux [38] of approximately 1.55×10^17^ photons·m^−2^·s^−1^. Intensity and shape of emitted light was measured with a spectrometer (USB4000, Ocean Optics, Dunedin, FL, USA). Yellow LEDs were the only source of visible light in the experimental room, so they were perceived by the insect as spots of light in the dark. At the end of each corridor, opposite arms were equipped with a pair of opposite LEDs to guarantee a more symmetrical stimulation environment. The rationale behind the use of two light stimulation points rather than one is that, in the latter setup a single LED light would produce two different photo-stimuli. The insect would see the light emitted directly by such LED, and the light reflected on the opposite wall of the arena; both emitted and reflected lights will differ in shape and intensity. By using two opposite LEDs in the same corridor, the insect was able to see two similar stimuli on each opposite side. The use of corridors reduced sources of reflected light, i.e., each corridor (‘x’ and ‘y’) channeled and separated the light reaching the insect from that reflected by the surroundings. The onset and cessation of light stimulus delivery to the CPB was synchronized with that of the chemical stimulus by use of a solenoid valve.

### Behavioral experiments

The responses of beetles were recorded in three consecutive 2-min periods: in the air stream alone (control), in the air stream plus other stimuli (test), and in the air stream after removal of the chemical and photic stimuli (end-control). Note that the eolic stimulation (air stream) was offered during control, test and end-control periods, but chemical and photic stimuli were only offered during the test period. In one series, each insect was stimulated with the male-produced aggregation pheromone (*S*)-CPB I in an air stream, in complete darkness. In another series, the insect was given the choice between the pheromone carried by the air current through a dark corridor and two opposite yellow LEDs placed at each end of the perpendicular corridor. In the third series, an insect was given the choice between a source of pheromone plus yellow LED placed above the tube (see Chemical stimulus) while the other three arms were provided with yellow LEDs. Experiments were performed during insect photophase, in a dark room at 25±0.5°C and 40±10% RH as measured by a hygro-thermometer (EA25, Extech Instruments, Waltham, MA, USA, accuracy: ±3% r.h. and ±1°C). Each of the three experimental series was run with 22 females and 22 males. Experiments started at least five minutes after the animal had been placed on the servosphere in darkness in order to allow it to acclimate to the experimental conditions. Although, this time was selected arbitrarily it served to standardize insect conditions. Insects that did not walk after 1 min were discarded (approximately <5%). Insects were used only once.

### Track analysis

The x-y co-ordinates provided by the servosphere at intervals of 0.1 s were merged in step-sizes of 10 units for efficient summarizing of the tracks [15], [39], [40]. This merger provided step-size intervals of 1 s which allowed the insect to move at least 50% of its length before recording its next position, taking into account that *L. decemlineata* mean length is approximately 1 cm and its average speed is approximately 0.5 cm/s. This step-size was large enough to reduce noise produced by insect movements such as wobbling while walking. Instantaneous speed and direction were computed from the position changes within each interval. Instantaneous turnings higher than 80° were removed; such values were considered noise after an analysis of video recordings. Insect walking tracks were reconstructed by plotting the cumulative addition of consecutive positions. To identify stops, 2 mm/s was considered the minimum speed *L. decemlineata* had to achieve to be considered walking; values below this speed were produced by other insect movements such as grooming of the antennae or legs. Length of the arc of the trajectory (referred here as walking distance) and average speed were calculated using a spreadsheet application with previously developed equations [41], [42]. Each arm within the dual choice arena was assigned with an arbitrary cone, 60° either side of each stimulus. We considered that a CPB chose a particular corridor (0°, 90°, 180° or 270° directions) when it walked into an assigned virtual cone (i.e. walking distance in the cone, see Fig. 1). In order to evaluate the effects of the experimental stimuli on behavior, percentage of walking distance in the cone and average speed were calculated for each 2-min track (i.e. for the control, test and end-control periods). Average velocities of female and male naïve insects were calculated by merging data of the three experiments control periods (n = 66 for each sex).

### Statistics

When residuals of our model followed a normal distribution and showed homoscedasticity, they were analyzed with one-way ANOVAs, Tukey-Kramer's post-hoc test and paired t-test; otherwise, indexes were analyzed with Kruskal-Wallis tests, Dunn's post-hoc test and Wilcoxon signed rank test. Equality of variance was tested with Levene's test and normality of the residuals of models was analyzed within each treatment with Shapiro-Wilk's W test. The data of the two control periods were compared by repeated-measures ANOVA or Friedman's ANOVA (when data deviated from normality), with the period as the repeated factor. The effect of the stimulation on orientation and speed was evaluated by calculating indexes representing the difference between the data obtained for the test and the control periods, for each experimental treatment. Post-hoc multiple comparisons were done following [43]. Results were considered significant at the 5% level; P significant levels were given in approximate values to facilitate reader comparisons. Statistical analyses were performed using R software version 2.12.2010-10-11, Vienna, Austria [44].
